# The Role of the Transcription Factor Nuclear Factor-kappa B in Thyroid Autoimmunity and Cancer

**DOI:** 10.3389/fendo.2018.00471

**Published:** 2018-08-21

**Authors:** Cesidio Giuliani, Ines Bucci, Giorgio Napolitano

**Affiliations:** Unit of Endocrinology, Department of Medicine and Sciences of Aging and Ce.S.I.-Me.T., University of Chieti–Pescara, Chieti, Italy

**Keywords:** NF-κB, thyroid autoimmunity, thyroid cancer, transcription factors, gene regulation, major histocompatibility complex, RET/PTC, BRAF^V600E^

## Abstract

Nuclear factor-kappa B (NF-κB) is a ubiquitous transcription factor that is involved in inflammatory and immune responses, as well as in regulation of expression of many other genes related to cell survival, proliferation, and differentiation. In mammals, NF-κB comprises five subunits that can bind to promoter regions of target genes as homodimers or heterodimers. The most common dimer is the p50/p65 heterodimer. The several combinations of dimers that can be formed contribute to the heterogeneous regulation of NF-κB target genes, and this heterogeneity is further increased by interactions of the NF-κB dimers with other transcription factors, such as steroid hormone receptors, activator protein-1 (AP-1), and cAMP response element binding protein (CREB). In the thyroid, several studies have demonstrated the involvement of NF-κB in thyroid autoimmunity, thyroid cancer, and thyroid-specific gene regulation. The role of NF-κB in thyroid autoimmunity was hypothesized more than 20 years ago, after the finding that the binding of distinct NF-κB heterodimers to the major histocompatibility complex class I gene is hormonally regulated. Further studies have shown increased activity of NF-κB in thyroid autoimmune diseases and in thyroid orbitopathy. Increased activity of NF-κB has also been observed in thyroid cancer, where it correlates with a more aggressive pattern. Of particular interest, mutation of some oncogenes or tumor suppressor genes involved in thyroid carcinogenesis results in constitutive activation of the NF-κB pathway. More recently, it has been shown that NF-κB also has a role in thyroid physiology, as it is fundamental for the expression of the main thyroid-specific genes, such as sodium iodide symporter, thyroid peroxidase, thyroglobulin, *Pax8*, and *TTF-1* (*NKX2-1*).

## Introduction

Nuclear factor-kappa B (NF-κB) was identified more than 30 years ago as a transcription factor that can stimulate the expression of the immunoglobulin κ light chain in B cells (1, 2). Further studies demonstrated that NF-κB binds to DNA as a dimer that is formed by the combination of several proteins that contain an N-terminal Rel homology domain, which is responsible for NF-κB DNA binding and dimerization. These proteins are members of the NF-κB family, and in mammals they include: subunit p50 and its precursor p105 (encoded by the *NF-* κ*B1* gene); subunit p52 and its precursor p100 (encoded by the *NF-*κ*B2* gene); subunit p65 (also called RelA); c-Rel; and RelB (Table 1) (2–4). The proteins p65, c-Rel, and RelB contain a C-terminal transcriptional activation domain (TAD) that confers the ability to activate gene expression, whereas p50 and p52 lack the TAD and can only stimulate transcription through formation of heterodimers with transcription factors that have a TAD. Otherwise, p50 and p52 can bind as homodimers and repress gene transcription by preventing binding to the DNA of dimers containing a TAD (5). In this Review, the term NF-κB is used to indicate the NF-κB family of transcription factors as a whole (as given in Table 1), whereas the specific proteins are indicated where appropriate.

**Table 1:** The NF-κB family members in mammals.

**Protein**	**Functional domains**	**Precursor**	**Gene symbol**
p50 subunit	RHD	p105	*NFkB1*
p52 subunit	RHD	p100	*NFkB2*
p65 subunit (RelA)	RHD, TAD	None	*RELA*
c-Rel	RHD, TAD	None	*REL*
RelB	RHD, TAD	None	*RELB*

*RHD, Rel homology domain; TAD, transcriptional activation domain See references (2–4)*.

Although first described in B lymphocytes, NF-κB is almost ubiquitous. It regulates the expression of hundreds of genes, most of which are involved in inflammatory and immune responses. Indeed, NF-κB has a fundamental role in lymphocyte development and activation, and it is essential for innate and adaptive immune responses. In addition to its role in inflammation and immunity, NF-κB regulates other genes involved in cell survival, proliferation, and differentiation (2, 4–8). The list of genes that can be regulated by NF-κB can be found at the following link: http://www.bu.edu/NF-kB/gene-resources/target-genes/.

**Figure 1:**
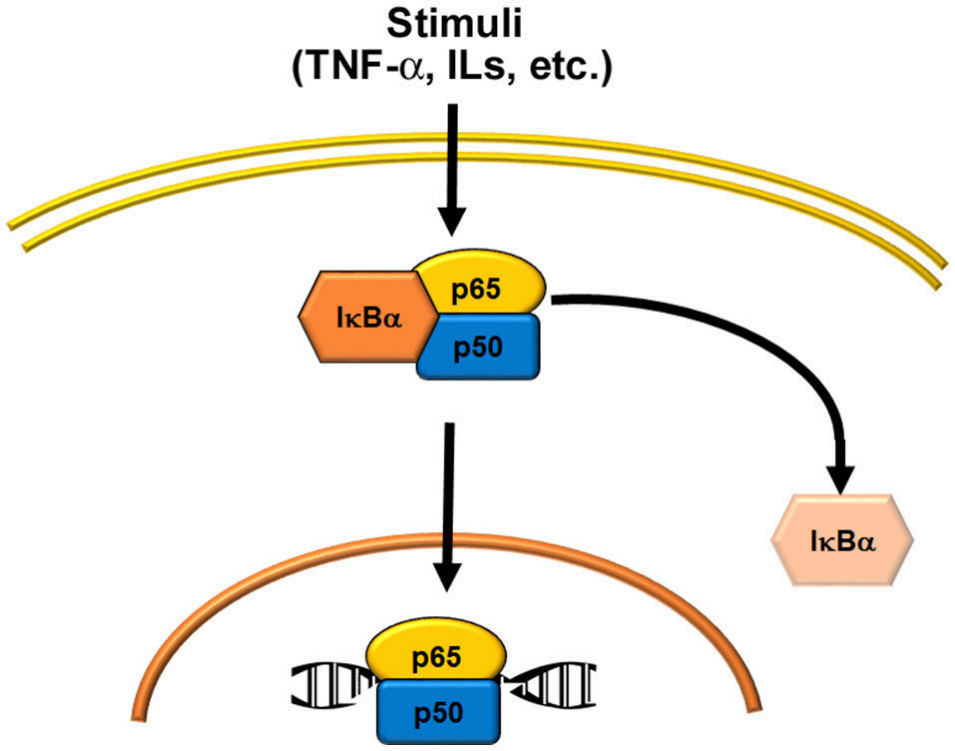
Schematic representations of NF-κB activation. Several stimuli (see Table 2) activate the classical or canonical pathway, where the p50/p65 heterodimer is the most common signal.

At its simplest, the mechanism by which NF-κB regulates transcription can be described as follows. In the resting state, NF-κB dimers are located in the cytoplasm in an inactive form, through their binding to inhibitory proteins known as inhibitors of κB (IκB). A wide range of stimuli can activate NF-κB through degradation of IκB (Table 2). This leads to translocation of the dimers into the nucleus, where they bind to a consensus sequence in the promoters of target genes (Figure 1). The first NF-κB dimer that was identified was the p50/p65 heterodimer (9), which is also the most abundant and widespread of the NF-κB dimers. In addition, several combinations have been described, both as homodimers, such as p65/p65, c-Rel/c-Rel, and p50/p50, and as heterodimers, such as p52/c-Rel, p50/c-Rel, RelB/p50, RelB/p52, p65/c-Rel, and p65/p52 (10).

**Table 2:** Main stimuli involved in NF-κB activation.

Pattern recognition receptors ligands (including PAMPs and DAMPs)
Cytokines (such as TNF-α, IL-1, IL-2, IL-17, IFNs)
T-cell receptor signals
B-cell receptor signals
Oxidative stress, hypoxia
Radiation (such as UV radiation, γ-radiation)
Mitogen-activated protein kinase signals

*DAMPs, damage-associated molecular patterns; IFN, interferon; IL, interleukin; PAMPs, pathogen-associated molecular patterns; TNF-α, tumor necrosis factor α; UV: ultraviolet*.

As indicated above, in unstimulated cells, NF-κB dimers are localized in the cytoplasm through their association with IκB proteins. However, it has been reported that the complex constituted by IκBα and the p50/p65 dimer can shuttle between the cytoplasm and the nucleus, although it remains transcriptionally inactive. Indeed, only after degradation of IκB proteins this dimer localizes to the nucleus and binds to DNA (2, 10). The IκB protein family is characterized by the presence of several ankyrin repeat (ANK) domains (i.e., five to seven) that are responsible for IκB binding to the NF-κB dimers. Several IκB proteins have been identified, including IκBα, IκBβ, IκBε, IκBκ, and Bcl-3. The precursor proteins p100 and p105 have several ANK domains in their C-terminal portions that work as IκB proteins, and therefore they are known as IκBδ and IκBγ, respectively (2, 5). IκBα and IκBβ are the best known members of the IκB family, as they are expressed in almost all tissues; conversely, the expression of IκBε, IκBζ, and Bcl-3 is restricted to hematopoietic cells (5, 10). The primary target of IκBα is the dimer p50/p65, whereas IκBβ is associated mainly with p65/c-Rel dimers. Several stimuli (Table 2) can activate NF-κB by triggering a signal cascade that ends in the phosphorylation of the IκB proteins, and their removal from the NF-κB dimer complex.

Two signaling pathways are involved in NF-κB activation: the classical or canonical pathway and the alternative or non-canonical pathway. The canonical pathway is the most common signaling involved in the activation of NF-κB. It is triggered by inflammatory cytokines, toll-like receptors, antigen receptors, and other stimuli, as given in Table 2. The canonical pathway activates the most common NF-κB dimers, which are formed by the p65, p50, c-Rel, and RelB subunits. The non-canonical pathway is involved in the activation of p100/RelB dimers, and it is induced by specific stimuli, such as B-cell activating factor (BAFF), lymphotoxin β, CD40 ligand, receptor activator of NF-κB ligand (RANKL), tumor necrosis factor (TNF)-like weak inducer of apoptosis (TWEAK), and TNF superfamily member 14 (also known as LIGHT) (11). The non-canonical NF-κB pathway is mainly involved in regulation of immune cell function and in bone remodeling (11).

As already mentioned, the precursor proteins p100 and p105 contain ANK domains and can function as IκB proteins. Usually, the precursor p105 is constitutively processed in cells, which produces the p50 subunit that binds to other NF-κB subunits to form dimers. However, dimers between p105 and other NF-κB subunits can also be formed; in this case, the ANK domains of the precursor protein function as an IκB protein, and their phosphorylation and degradation activate p50-containing dimers (12). Instead, precursor p100 is processed only after stimulation of the non-canonical pathway, which generates p52-containing dimers.

Activated NF-κB dimers bind to specific DNA binding sites (called κB sites) that are located in the promoter regions of the target genes. The consensus κB site has a partial palindromic sequence, 5′-GGGRNWYYCC-3′, where R is any purine (A or G), N is any nucleotide, W is A or T, and Y is any pyrimidine (C or T) (2). The constitution of different NF-κB dimers allows the regulation of distinct sets of genes. The mechanism of transcriptional regulation by NF-κB is complex and remains not fully understood (13). The several combinations of dimers that can be formed are fundamental for selective regulation of the target genes. Indeed, differences in DNA binding affinities have been reported between the different NF-κB subunits and the variant κB sites of target genes. Some dimers, such as c-Rel, p50, and Rel A homodimers, can bind κB sites that contain only half of the site consensus sequence (14). Also, the same NF-κB dimer can have different effects on gene transcription based on small differences in the κB site. An example is seen for the p52/Bcl-3 dimer, which activates transcription in genes with a κB site that contains the nucleotides G/C in the central position, whereas it represses transcription in genes where the nucleotides A/T are located in the central position of the κB site (15). Furthermore, NF-κB dimers can interact with several transcriptional coactivators (e.g., interactions between p65 and the coactivators p300/CBP) and with chromatin complexes. These interactions are particularly important for NF-κB dimers that lack a TAD; i.e., p50 and p52. Indeed, it has been reported that p50 and p52 homodimers can stimulate gene transcription through their interactions with nuclear IkB proteins such as Bcl-3 and IkB ζ, which function as coactivators. Furthermore, p50 and p52 homodimers can repress gene expression through interactions with histone deacetylases. A further mechanism that contributes to heterogeneous regulation of target genes by NF-κB is through its physical interactions with steroid hormone receptors, and it can also form heterodimers with different families of transcription factors, such as activator protein-1 (AP-1) and cAMP response element binding protein (CREB) (16–20).

## NF-κB and the thyroid

The presence of NF-κB in thyrocytes was reported more than 20 years ago, both in a human thyroid carcinoma cell line (21) and in a non-transformed rat thyroid cell line (17). Thenceforth, several studies have demonstrated the involvement of NF-κB in thyroid autoimmunity, thyroid cancer, and thyroid-specific gene regulation (7, 21–25). It is worth to remark that the role of NF-κB in regulating the expression of the thyroid-specific genes has been demonstrated several years after the discovery of its involvement in thyroid autoimmunity and cancer.

### NF-κB and thyroid autoimmunity

**Figure 2:**
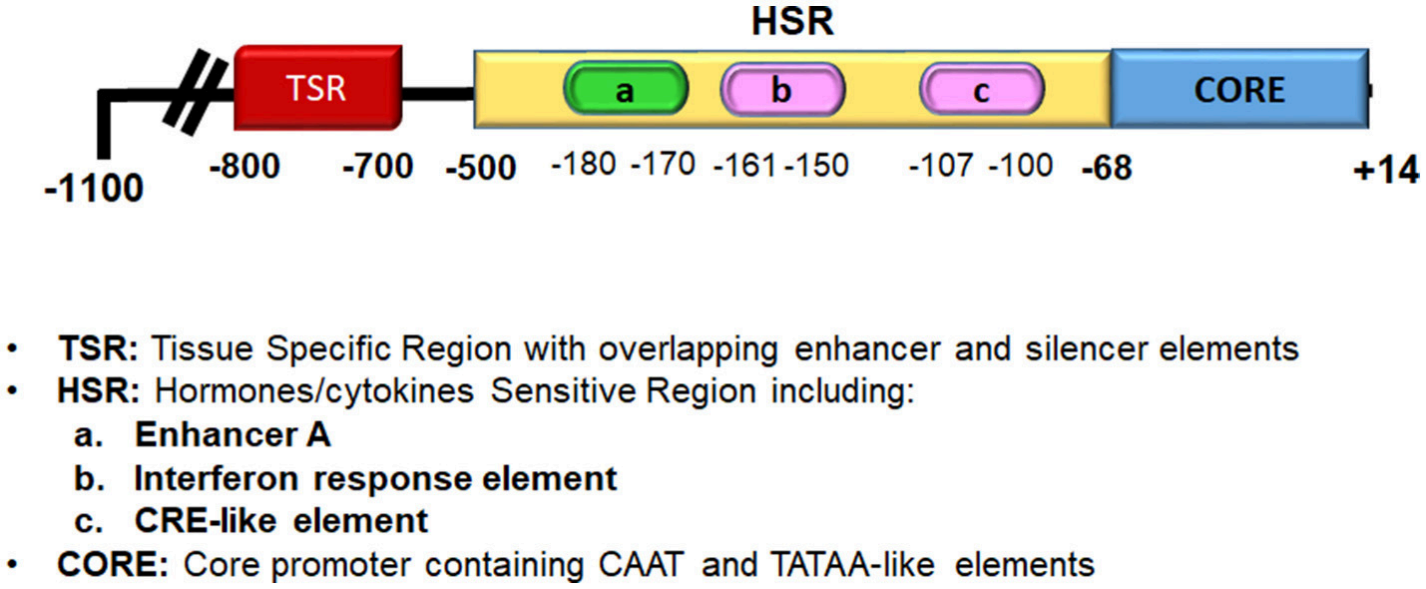
Diagrammatic representation of the MHC class I gene promoter.

NF- κB has a fundamental role in both innate and adaptive immune responses. Indeed, NF-κB is one of the main transcription factors that is activated by pattern recognition receptors (PRRs), cytokines receptors, and lymphocyte receptors; therefore, it is not surprising that several studies have demonstrated its involvement in the development of autoimmune diseases (22). Of note, thyroid cells have functional PRRs, such as toll-like receptors (TLRs) and RIG-like receptors, that respond to various pathogen-associated molecular patterns (PAMPs) or damage-associated molecular patterns (DAMPs), to induce the production of several cytokines and chemokines (26). On this basis, it has been hypothesized that several insults to thyrocytes through the production of PAMPs or DAMPs can trigger an innate immune response, and eventually make thyrocytes behave as antigen-presenting cells (APCs), which can recruit and activate lymphocytes, and hence initiate an autoimmune response (26–28). An intriguing observation is that in rat thyroid cells in continuous culture, the FRTL-5 cells, there is a hormonal regulation of NF-κB activation, and of its binding to DNA. Indeed, studies on the major histocompatibility complex (MHC) class I gene in thyrocytes have shown that its expression is regulated by several hormones and growth factors through the regulation of NF-κB binding to the MHC class I promoter. Many studies have demonstrated that the main hormones involved in the regulation of thyroid growth and function decrease MHC class I expression, which include thyroid-stimulating hormone (TSH), insulin/ insulin-like growth factor (IGF)-I and hydrocortisone, (17, 29–31). MHC class I overexpression, as well as MHC class II aberrant expression, on non-immune cells is a feature of autoimmune diseases and thyroid autoimmunity (27, 32–37). The hormonal regulation of MHC molecules in thyroid cells is considered important for the suppression of autoimmunity during hormonally induced changes in cellular growth and function, which results in enhanced expression of potential thyroid autoantigens, such as thyroglobulin, thyroid peroxidase, and the TSH receptor (TSHR). Of note, MHC class I expression is also decreased by iodide, phorbol esters, transforming growth factor (TGF)-β, and methimazole, whereas it is increased by interferon (IFN)-α and IFN-γ, thymosin-α1, and high levels of glucose (17, 38–42). It must be emphasized that the regulation of MHC class I gene expression by these hormones and growth factors involves the binding of NF-κB dimers to the enhancer A region of the MHC class I promoter (Figure 2). The enhancer A region is located in a “hormone-sensitive region” of the MHC class I promoter (i.e., −500 to −68 bp), and this region is responsible for the regulation of MHC class I expression by hormones, cytokines, chemokines, and drugs (33, 35, 40). The enhancer A sequence (5′-GGGGAGTCCCC-3′) that spans nucleotides −180 bp to −170 bp is a palindromic variant of the κB consensus site, and it can bind NF-κB dimers (43). Its core sequence, GGGGA, is common to κB sites from other genes, such as that of the immunoglobulin κ light chain (38). Using electrophoretic mobility shift assays, it has been demonstrated that in thyrocytes, the enhancer A sequence can bind several NF-κB dimers. The first dimer identified, named Mod-1, is an unusual heterodimer that comprises the p50 subunit of NF-κB and fra-2, a transcription factor member of the AP-1 family (17). Modulation of Mod-1 binding affects MHC class I expression. Indeed, increased Mod-1 binding to enhancer A results in increased expression of the promoter activity, whereas the opposite is seen when there is decreased Mod-1 binding. Enhancer A also binds the classic NF-κB heterodimer p50/p65, which has an opposite effect compared to Mod-1 (38). In brief, several factors regulate MHC class I expression through modification of the binding of the heterodimers Mod-1 and p50/p65 to enhancer A. As an example, iodide, phorbol esters, and TGF-β decrease MHC class I gene transcription through inhibition of Mod-1 binding, while they allow p50/p65 binding (38, 39). Conversely, factors such as glucose and thymosin-α1, which activate MHC class I gene transcription, act through increasing Mod-1 binding and decreasing p50/p65 binding to enhancer A (41, 42). Further studies have demonstrated that NF-κB also interacts with a dominant regulatory element of the MHC class I promoter that is located between −800 bp and −700 bp, and which regulates tissue-specific transcription through overlapping enhancer and silencer elements. The binding of a complex in this region has been observed, which contains the p65 subunit of NF-κB and c-jun (31, 39). Of note, the two primary regions involved in the regulation of MHC class I transcription in thyroid cells interact with different members of the same family of transcription factors, NF-κB and AP-1, and both these factors are involved in the signaling of PPRs and cytokine receptors.

The data obtained on the regulation of the MHC class I gene are not restricted to this gene, as they can also be applied on the regulation of all of the thyroid genes where transcription is modulated by NF-κB (44–47). In this regard, the intercellular adhesion molecule (ICAM)-1 gene is hormonally regulated in FRTL-5 cells, and this regulation involves NF-κB, similarly to that observed for the MHC class I gene (44).

A conclusion that comes from these data is that different NF-κB dimers can modify the expression of the target genes. Therefore, an observation that is of significant interest is that in thyroid cells, stimulation of TSHR by TSH or stimulating antibodies to TSHR (TSAbs) can modify the composition of the NF-κB dimers activated by TNF-α. Indeed, in the absence of TSH, TNF-α treatment activates only the p50 homodimers, whereas in the presence of TSH, there is also activation of the p50/p65 heterodimers, which results in the modification of target gene expression (48).

Further progress on the understanding of the role of NF-κB in thyroid autoimmunity was derived from studies on CD40 signaling in thyroid cells. CD40 is a member of the TNF family that is expressed in immune cells and some non-immune cells, including thyroid cells. CD40 overexpression on thyroid cells has been associated with the development of autoimmunity (49). A recent study has showed that CD40 activation up-regulates expression of the p65 and p52 subunits of NF-κB in human primary thyroid cell cultures from Graves' patients, which indicates an involvement of both the canonical and noncanonical NF-κB pathways in CD40 signaling in Graves' disease (50).

Involvement of NF-κB activation is also seen in the pathogenesis of Graves' orbitopathy. Graves' orbitopathy is characterized by infiltration of the orbit by fibrocytes, which produce proinflammatory cytokines and induce an inflammatory reaction. Cytokine production by fibrocytes is stimulated by TSH and TSAbs, which interact with a receptor complex that is formed by the TSHR and the IGF-I receptor. Stimulation of this receptor complex results in activation of both the Akt and NF- κB pathways (51). As previously reported for thyroid cells, CD40 signaling can activate NF-κB on these orbit fibrocytes (52). At present, which NF-κB dimers are activated on orbital fibrocytes is not known.

### NF-κB and thyroid cancer

**Figure 3:**
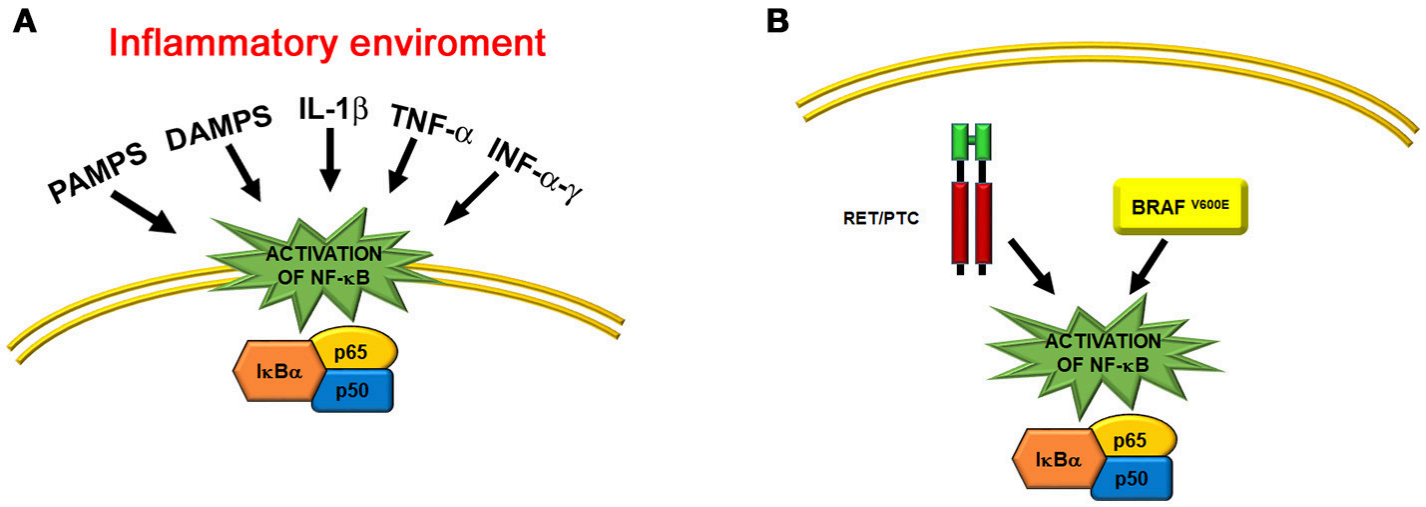
Schematic representations of the two main mechanisms that activate NF-κB in thyroid cells. **(A)** Activation of the NF-κB pathway in an inflammatory environment, such as Hashimoto's thyroiditis. Some of the main factors that act throughout transmembrane and cytosolic receptors are indicated. **(B)** Activation of the NF-κB pathway by thyroid oncogenes, such as the cytosolic RET/PTC rearranged protein and the cytosolic BRAF^V600E^ mutated kinase.

A large number of studies on the role of NF-κB in the pathogenesis of cancer followed the observations that NF-κB transcription factors have homology with the avian oncogene *v-REL*, which causes reticuloendotheliosis and lymphoma in poultry (53), and that human *c-REL* can induce transformation of primary chicken spleen cells (54). Abnormal activation of NF-κB has been associated not only with lymphoid malignancies (55), but also with tumors of epithelial origin, including thyroid cancer (23, 56). This is not surprising given the well-recognized connection between inflammation and tumor development (57–59). NF-κB promotes the production of cytokines, chemokines, growth factors, and other molecules that constitute the tumor microenvironment. Furthermore, NF-κB increases the expression of anti-apoptotic genes, such as *BCL2*, and mitogenic genes, such as c-*MYC* and cyclin D1 (23, 56). Therefore, NF-κB activation makes tumor cells resistant to pro-apoptotic stimuli, as observed for TGF-β apoptotic effects in thyroid cancer cells (60). Activation of NF-κB in tumors can arise from both a response to classical inflammatory stimuli, such as infectious and physical or chemical agents (Figure 3A), and the result of oncogene activation (Figure 3B) (58). A typical example of the latter is the *RET* oncogene, which is involved in several cancer types, including thyroid cancer (61). Indeed, activating mutations of the *RET* gene are responsible for medullary thyroid carcinomas, and RET/PTC rearrangements are associated with some 5 to 25% of thyroid papillary carcinomas (62). Of interest, activating mutations of the *RET* proto-oncogene cause constitutive activity of NF-κB, and this process is important for *RET*-mediated carcinogenesis (63, 64). Indeed, abnormal expression of the RET/PTC1 oncogene in primary cultures of normal human thyrocytes is sufficient to induce the expression of a large panel of genes that can be activated by NF-κB and are involved in inflammation, among which there are the colony-stimulating factors, interleukin-1β, and cyclooxygenase 2 (65).

For thyroid cancers, constitutive increased DNA-binding activity of NF-κB was reported for the first time about 20 years ago, in a series of seven human thyroid carcinoma cell lines that included papillary, follicular, and anaplastic carcinomas (66). In particular, the increased binding activity was associated with overexpression of the p65 subunit, whereas the p50 subunit was not overexpressed in the anaplastic carcinoma cell lines, and was increased only in cell lines derived from papillary or follicular carcinoma, and to a lesser extent than for p65. The role of the p65 subunit was confirmed by the observation that inhibition of p65 expression using a specific antisense oligonucleotide reduced the growth of cell lines and their colony formation in agar. Successively, several studies showed that activation of NF-κB is a key merging point of distinct transforming signals that are involved in thyroid carcinogenesis, which besides the *RET* oncogene, included others such as BRAF^V600E^ mutation, *PPAR*γ insufficiency, and *PTEN* inactivation (67–69). Indeed, induction of a mutated form of the *BRAF* gene (e.g., the BRAF^V600E^ mutation) in models of follicular and papillary thyroid cancer cell lines resulted in NF-κB activation, with involvement of the p65/p50 heterodimer (67). This NF-κB activation resulted in apoptotic resistance and increased invasiveness of these cells, as a consequence of increased expression of anti-apoptotic molecules and matrix metalloproteinases. Furthermore, in a mouse model of thyroid cancer, inactivation of the *PTEN* or *PPAR*γ genes gave rise in both cases to a more aggressive form of cancer, which was associated with NF-κB overactivation (68, 69). As well as the experimental data, NF-κB activation has also been shown in human thyroid cancer tissues (23, 70–72). A first study that was performed on 10 specimens of thyroid follicular carcinoma investigated the p65 subunit selectively and showed its constitutive activation and translocation to the nucleus (70). A following study showed increased nuclear expression of the p65 subunit in about 75% of a larger sample of papillary carcinomas. Interestingly, these tumors were associated with a significantly higher frequencies of aggressive features, such as extrathyroidal extension and lymph node metastasis (71). The important role of NF-κB in the pathogenesis of thyroid cancer was also confirmed by the efficacy of several inhibitors of NF-κB activation in the counteracting of its effects on growth and invasiveness in experimental models (23, 73–76).

However, very few studies have evaluated the binding of the distinct NF-κB dimers to DNA in thyroid cancers. Most of the studies performed both *in vitro* and *in vivo* have investigated NF-κB activation using only antibodies against the p65 subunit. A few studies have also investigated the p50 subunit, which showed that the p50/p65 heterodimer is the main complex involved in DNA binding (66, 74, 77). However, no data is available so far concerning the involvement of other NF-κB dimers. There is also no information available on the binding of distinct dimers to the promoters of specific genes involved in thyroid carcinogenesis. Therefore, a working hypothesis would investigate the distinct dimer combinations involved in binding to the promoter region of genes activated in thyroid carcinogenesis, as described above for the MHC class I gene in FRTL-5 cells. Moreover, more data are needed on the interactions between NF-κB and other transcription factors, as some studies have suggested that this is an important step in thyroid carcinogenesis and it can be used as a molecular target for therapy (75, 76). On this point, an experimental study has shown that the efficacy of triptolide in inhibition of the growth and invasiveness of an anaplastic thyroid cancer cell line is due to its blocking of the association between the p65 subunit of NF-κB and CBP/P300 (75). Similarly, the retinoid X receptor agonist bexarotene that has been used to treat metastatic differentiated thyroid cancer in clinical trials, represses NF-κB activation in follicular cancer cells through inhibition of the interaction between p300 and the p65 subunit (76).

### NF-κB and thyroid-specific gene regulation

The findings that NF-κB is involved in the regulation of thyroid-specific genes is of particular interest (45–47). In a study of the effects of lipopolysaccharide on *NIS* gene expression in FRTL-5 cells, Nicola et al. (45) defined a κB binding site in the upstream enhancer region of the *NIS* promoter (NUE). They also observed that this site binds the p65 subunit of NF-κB, and that this subunit acts in synergy with transcription factor Pax8 for the promotion of gene transcription. Indeed, a physical interaction between p65 and Pax8 was reported. These data confirm the involvement of heterodimers between NF-κB subunits and other transcription factors in the regulation of gene expression in thyroid cells, as discussed above regarding the expression of the MHC class I gene in FRTL-5 cells. Subsequently, the same group showed that the p65 subunit is also involved in the regulation of *TPO* gene expression by lipopolysaccharide (46). A recent study further highlighted the role of NF-κB in thyroid physiology (25). Indeed, a mouse model bearing a thyroid-specific knock-out of the NF-κB essential modulator (NEMO) gene, which is fundamental for IκB phosphorylation, developed hypothyroidism and thyroid hypoplasia due to massive apoptosis. Of further interest, in this model there was a reduced expression of the thyroid specific genes *NIS, TPO, TG, Pax8*, and *TTF-1* (*NKX2-1*). These data are very important, as they demonstrate that as well as its involvement in thyroid autoimmunity and cancer, NF-κB is also fundamental for the regulation of genes related to normal thyroid function (25, 78).

## Conclusions

Since its discovery more than 30 years ago, NF-κB has become a pillar of cellular biology. A large number of studies have shown its fundamental roles in regulating cellular functions. The importance of this can be perceived by considering the great number of genes that can be regulated by NF-κB. For the thyroid, after the first studies that showed the role of NF-κB in the regulation of MHC genes, several observations underlined its further role as a common target of the distinct PRRs pathways, which confirmed its involvement in the pathogenesis of thyroid autoimmunity. Not surprisingly, given the relationship between thyroid autoimmunity and cancer, NF-κB is also involved in thyroid carcinogenesis, and it is considered a potential pharmacological target for new therapies against the most aggressive types of thyroid cancers. As well as these observations on the role of NF-κB in thyroid pathology, recent studies of great relevance have correlated NF-κB with normal thyroid growth and function. These data indicate how much more we have to learn about the function of NF-κB in both thyroid physiology and physiopathology. As some studies have suggested, an important issue that remains largely unexplored relates to the binding of the distinct NF-κB subunits to DNA, and particularly the different combinations of homodimers and heterodimers involved, including those with other families of transcription factors. Therefore, more studies are needed to understand the physiological role of NF-κB in thyrocytes and its dysfunction in thyroid pathology.

## Author contributions

CG: substantial contributions to the conception and design of the work; drafting the work; final approval of the version to be published; and agreement to be accountable for all aspects of the work in ensuring that questions related to the accuracy or integrity of any part of the work are appropriately investigated and resolved. IB: substantial contributions to the design of the work; revising the work critically for important intellectual content; final approval of the version to be published; and agreement to be accountable for all aspects of the work in ensuring that questions related to the accuracy or integrity of any part of the work are appropriately investigated and resolved. GN: substantial contributions to the conception of the work; revising it critically for intellectual content; final approval of the version to be published; and agreement to be accountable for all aspects of the work.

### Conflict of interest statement

The authors declare that the research was conducted in the absence of any commercial or financial relationships that could be construed as a potential conflict of interest.

The reviewer SF and handling editor declared their shared affiliation at time of review.

## References

[1] Sen R Baltimore D. Multiple nuclear factors interact with the immunoglobulin enhancer sequences. Cell (1986) 46:705–16. 10.1016/0092-8674(86)90346-6 3091258

[2] Zhang Q Lenardo MJ Baltimore D Thirty years of NF-κB: a blossoming of relevance to human pathobiology. Cell (2017) 168:37–57. 10.1016/j.cell.2016.12.012 28086098 PMC5268070

[3] Napetschnig J Wu H. Molecular basis of NF-κB signaling. Annu Rev Biophys. (2013) 42:443–68. 10.1146/annurev-biophys-083012-130338 23495970 PMC3678348

[4] Hinz M Scheidereit C. The IκB kinase complex in NF-κB regulation and beyond. EMBO Rep. (2013) 15:46–61. 10.1002/embr.201337983 24375677 PMC4303448

[5] Hayden TH Gosh S. NF-κB, the first quarter-century: remarkable progress and outstanding questions. Genes Dev. (2012) 26:203–34. 10.1101/gad.183434.111 22302935 PMC3278889

[6] Gilmore TD Wolenski FS. NF-κB: where did it come from, and why? Immunol Rev. (2012) 246:14–35. 10.1111/j.1600-065X.2012.01096.x 22435545

[7] Giuliani C Napolitano G Bucci I Montani V Monaco F NF-κB transcription factor: role in the pathogenesis of inflammatory autoimmune and neoplastic diseases and therapy implications. Clin Ter. (2001) 152:249–53. 11725618

[8] Santoro MG Rossi A Amici C. NF-κB and virus infection: who controls whom? EMBO J. (2003) 22:2552–60. 10.1093/emboj/cdg267 12773372 PMC156764

[9] Baeuerle PA Baltimore D A 65-kD subunit of active NF-κB is required for inhibition of NF-κB by IκB. Genes Dev. (1989) 3:1689–98. 10.1101/gad.3.11.1689 2691328

[10] Oeckinghaus A Gosh S. The NF-κB family of transcription factors and its regulation. Cold Spring Harb Perspect Biol. (2009) 1:000034. 10.1101/cshperspect.a000034 20066092 PMC2773619

[11] Cildir C Low KC Tergaonkar V. Noncanonical NF-κB signaling in health and disease. Trends Mol Med. (2016) 22:414–29. 10.1016/j.molmed.2016.03.002 27068135

[12] Kravtsova-Ivantsiv Y Cohen S Ciechanover A. Modification by single ubiquitin moieties rather than polyubiquitination is sufficient for proteasomal processing of the p105 NF-κB precursor. Mol Cell (2009) 33:496–504. 10.1016/j.molcel.2009.01.023 19250910

[13] Smale ST. Dimer-specific regulatory mechanisms within the NF-κB family of transcription factors. Immunol Rev. (2012) 246:193–204. 10.1111/j.1600-065X.2011.01091.x 22435556

[14] Siggers T Chang AB Teixeira A Wong D Williams KJ Ahmed B Principle of dimer-specific gene regulation revealed by a comprehensive characterization of NF-κB family DNA binding. Nat Immunol. (2012) 13:95–102. 10.1038/ni.2151 PMC3242931 22101729

[15] Wang VY-F Huang W Asagiri M Spann N Hoffmann A Glass C The transcriptional specificity of NF-κB dimers is coded within the κB DNA response element. Cell Rep. (2012) 2:824–39. 10.1016/j.celrep.2012.08.042 23063365 PMC4167904

[16] Stein B Baldwin AS Ballard DW Greene WC Angel P Herrlich P Cross-coupling of NF-κB p65 and Fos/Jun transcription factors produces potentiated biological function. EMBO J. (1993) 12:3879–91. 8404856 10.1002/j.1460-2075.1993.tb06066.x PMC413671

[17] Giuliani C Saji M Napolitano G Palmer LA Taniguchi S-I Shong M Hormonal modulation of major histocompatibility complex class I gene expression involves an enhancer A-binding complex consisting of fra-2 and the p50 subunit of NF-κB. J Biol Chem. (1995) 270:11453–62. 10.1074/jbc.270.19.11453 7744783

[18] Yao C Purwanti N Karabasil MR Azlina A Javkhlan P Hasegawa T. Potential down-regulation of salivary gland AQP5 by LPS via cross-coupling of NF-κB and p-c-Jun/c-Fos. Am J Pathol. (2010) 177:724–34. 10.2353/ajpath.2010.090282 20522648 PMC2913374

[19] McKay LI Cidlowski JA Cross-talk between nuclear factor-κB and the steroid hormone receptors: mechanisms of mutual antagonism. Mol Endocrinol. (1998) 12:45–66. 10.1210/mend.12.1.0044 9440809

[20] Ling J Kumar R Cross-talk between NF-κB and glucocorticoid signaling: a potential target of breast cancer therapy. Cancer Lett. (2012) 322:119–66. 10.1016/j.canlet.2012.02.033 22433713

[21] Pang X-P Ross NS Park M Juillard GJF Stanley TM Hershman JM Tumor necrosis factor-α activates nuclear factor κB and induces manganous superoxide dismutase and phosphodiesterase mRNA in human papillary thyroid carcinoma cells. J Biol Chem. (1992) 267:12826–30. 1320006

[22] Kurylowicz A Nauman J. The role of nuclear factor-κB in the development of autoimmune diseases: a link between genes and environment. Acta Biochim Pol. (2008) 55: 629–47. 19081854

[23] Pacifico F Leonardi A. Role of NF-κB in thyroid cancer. Mol Cell Endocrinol. (2010) 321:29–35. 10.1016/j.mce.2009.10.010 19879919

[24] Zeligs KP Neuman MK Annunziata CM. Molecular pathways: the balance between cancer and the immune system challenges the therapeutic specificity of targeting nuclear factor-κB signaling for cancer treatment. Clin Cancer Res. (2016) 22:4302–08. 10.1158/1078-0432.CCR-15-1374 27422962 PMC5010470

[25] Reale C Iervolino A Scudiero I Ferravante A D'Andrea LE Mazzone P. NF-κB essential modulator (NEMO) is critical for thyroid function. J Biol Chem. (2016) 291:5765–73. 10.1074/jbc.M115.711697 26786105 PMC4786713

[26] Kawashima A Yamazaki K Hara T Akama T Yoshihara A Sue M. Demonstration of innate immune responses in the thyroid gland: potential to sense danger and a possible trigger for autoimmune reactions. Thyroid (2013) 23:477–87. 10.1089/thy.2011.0480 23234343 PMC3610444

[27] Luo Y Yoshihara A Oda K Ishido Y Suzuki K. Excessive cytosolic DNA fragments as a potential trigger of Graves' disease: an encrypted message sent by animal models. Front Endocrinol. (2016) 7:144. 10.3389/fendo.2016.00144 27895620 PMC5107990

[28] Harii N Lewis CJ Vasko V McCall K Benavides-Peralta U Sun X. Thyrocytes express a functional toll-like receptor 3: overexpression can be induced by viral infection and reversed by phenylmethimazole and is associated with Hashimoto's autoimmune thyroiditis. Mol Endocrinol. (2005) 19:1231–50. 10.1210/me.2004-0100 15661832

[29] Saji M Moriarty J Ban T Singer DS Kohn LD. Major histocompatibility complex class I gene expression in rat thyroid cells is regulated by hormones methimazole and iodide as well as interferon. J Clin Endocrinol Metab. (1992) 75:871–8. 1381373 10.1210/jcem.75.3.1381373

[30] Saji M Shong M Napolitano G Palmer LA Taniguchi S-I Ohmori M. Regulation of major histocompatibility complex class I gene expression in thyroid cells. J Biol Chem. (1997) 272:20096–107. 10.1074/jbc.272.32.20096 9242683

[31] Giuliani C Saji M Bucci I Fiore G Liberatore M Singer DS. Transcriptional regulation of major histocompatibility complex class I gene by insulin and IGF-I in FRTL-5 thyroid cells. J Endocrinol. (2006) 189:605–15. 10.1677/joe.1.06486 16731791

[32] Mozes E Kohn LD Hakim F Singer DS. Resistance of MHC class I-deficient mice to experimental systemic lupus erythematosus. Science (1993) 261:91–3. 10.1126/science.8316860 8316860

[33] Singer DS Mozes E Kirshner S Kohn LD. Role of MHC class I molecules in autoimmune disease. Crit Rev Immunol. (1997) 17:463–8. 9419433

[34] Ito T Meyer KC Ito N Paus R. Immune privilege and the skin. Curr Dir Autoimmun. (2008) 10:27–52. 10.1159/000131412 18460879

[35] René C Lozano C Eliaou JF. Expression of classical HLA class I molecules: regulation and clinical impacts. HLA (2016) 87:338–49. 10.1111/tan.12787 27060357

[36] Richardson SJ Rodriguez-Calvo T Gerling IC Mathews CE Kaddis JS Russell MA. Islet cell hyperexpression of HLA class I antigens: a defining feature in type 1 diabetes. Diabetologia (2016) 59:2448–58. 10.1007/s00125-016-4067-4 27506584 PMC5042874

[37] Gianfran C Pisapia L Picascia S Strazzullo M Del Pozzo G Expression level of risk genes of MHC class II is a susceptibility factor for autoimmunity: new insights. J Autoimmun. (2018) 89:1–10. 10.1016/j.jaut.2017.12.016 29331322

[38] Taniguchi S-I Shong M Giuliani C Napolitano G Saji M Montani V Iodide suppression of major histocompatibility class I gene expression in thyroid cells involves enhancer A and the transcription faactor NF-κB. Mol Endocrinol. (1998) 12:19–33. 10.1210/mend.12.1.0052 9440807

[39] Napolitano G Montani V Giuliani C Di Vincenzo S Bucci I Todisco V Transforming growth factor-?1 down-regulation of major histocompatibility complex class I in thyrocytes: coordinate regulation of two separate elements by thyroid-specific as well as ubiquitous transcription factors. Mol Endocrinol. (2000) 14:486–505. 10.1210/mend.14.4.0454 10770487

[40] Giuliani C Bucci I Montani V Singer DS Monaco F Kohn LD. Regulation of major histocompatibility complex gene expression in thyroid epithelial cells by methimazole and phenylmethimazole. J Endocrinol. (2010) 204:57–66. 10.1677/JOE-09-0172 19837722 PMC6310398

[41] Napolitano G Bucci I Giuliani C Massafra C Di Petta C Devangelio E. High glucose levels increase major histocompatibility complex class I gene expression in thyroid cells and amplify interferon-γ action. Endocrinology (2002) 143:1008–17. 10.1210/endo.143.3.8674 11861526

[42] Giuliani C Napolitano G Mastino A Di Vincenzo S D'Agostini C Grelli S. Thymosin-α1 regulates MHC class I expression in FRTL-5 cells at transcriptional level. Eur J Immunol. (2000) 30:778–86. 10.1002/1521-4141(200003)30:3 < 778::AID-IMMU778>3.0.CO;2-I 10741392

[43] Baldwin AS Sharp PA Two transcription factors NF-κB and H2TF1 interact with a single regulatory sequence in the class I major histocompatibility complex promoter. Proc Natl Acad Sci USA. (1988) 85:723–7. 10.1073/pnas.85.3.723 3422454 PMC279627

[44] Park ES You SH Kim H Kwon OY Ro HK Cho BY. Hormone-dependent regulation of intercellular adhesion molecule-1 gene expression: cloning and analysis of 5'-regulatory region of rat intercellular adhesion molecule-1 gene in FRTL-5 rat thyroid cells. Thyroid (1999) 9:601–12. 10.1089/thy.1999.9.601 10411124

[45] Nicola JP Nazar M Mascanfroni ID Pellizas CG Masini-Repiso AM. NF-κB p65 subunit mediates lipopolysaccharide-induced Na(+)/I(-) symporter gene expression by involving functional interaction with the paired domain transcription factor Pax8. Mol Endocrinol. (2010) 24:1846–62. 10.1210/me.2010-0102 20667985 PMC5417406

[46] Nazar M Nicola JP Velez ML Pellizas CG Masini-Repiso AM. Thyroid peroxidase gene expression is induced by lipopolysaccharide involving nuclear factor (NF)-κB p65 subunit phosphorylation. Endocrinology (2012) 153:6114–25. 10.1210/en.2012-1567 23064013

[47] Nicola JP Peyret V Nazar M Romero JM Lucero AM Montesinos Mdel M. S-Nitrosylation of NF-κB p65 inhibits TSH-induced Na(+)/I(-) symporter expression. Endocrinology (2015) 156:4741–54. 10.1210/en.2015-1192 26587909

[48] Kikumori T Kambe F Nagaya T Funahashi H Seo H Thyrotropin modifies activation of nuclear factor kB by tumour necrosis factor α in rat thyroid cell line. Biochem J. (2001) 354:573–79. 10.1042/bj3540573 11237861 PMC1221688

[49] Kayes T Fang Y Yu S Downey E Wang S Braley-Mullen H. Agonistic anti-CD40 induces thyrocyte proliferation and promotes thyroid autoimmunity by increasing CD40 expression on thyroid epithelial cells. J Immunol. (2013) 190:3928–38. 10.4049/jimmunol.1202929 23509363 PMC3622184

[50] Lee HJ Lombardi A Stefan M Li CW Inabnet WBIII Owen RP CD40 signaling in Graves' disease is mediated through canonical and noncanonical thyroidal nuclear factor κB activation. Endocrinology (2017) 158:410–8. 10.1210/en.2016-1609 27929668 PMC5413074

[51] Chen H Shan SJC Mester T Wei Y-H Douglas RS TSH-mediated TNF? production in human fibrocytes is inhibited by teprotumumab an IGF-1R antagonist. PLoS ONE (2015) 10:e0130322 10.1371/journalpone013032. 26087256 PMC4472723

[52] Wu T Mester T Gupta S Sun F Smith TJ Douglas RS. Thyrotropin and CD40L stimulate interleukin-12 expression in fibrocytes: implications for pathogenesis of thyroid-associated ophthalmopathy. Thyroid (2016) 26:1768–77. 10.1089/thy.2016.0243 27612658 PMC5175425

[53] Gilmore TD. Multiple mutations contribute to the oncogenicity of the retroviral oncoprotein v-Rel. Oncogene (1999) 18:6925–37. 10.1038/sj.onc.1203222 10602467

[54] Gilmore TD Cormier C Jean-Jacques J Gapuzan ME. Malignant transformation of primary chicken spleen cells by human transcription factor c-Rel. Oncogene (2001) 20:7098–103. 10.1038/sj.onc.1204898 11704834

[55] Lim KH Yang Y Staudt LM. Pathogenetic importance and therapeutic implications of NF-κB in lymphoid malignancies. Immunol Rev. (2012) 246:359–78. 10.1111/j.1600-065X.2012.01105.x 22435566 PMC4094296

[56] Pires BRB Silva RCMC Ferreira GM Abdelhay E. NF-κB: two sides of the same coin. Genes (2018) 9:E24. 10.3390/genes9010024 29315242 PMC5793177

[57] Balkwill F Mantovani A. Inflammation and cancer: back to Virchow? Lancet (2001) 357:539–45. 10.1016/S0140-6736(00)04046-0 11229684

[58] Mantovani A Allavena P Sica A Balkwill F. Cancer-related inflammation. Nature (2008) 454:436–44. 10.1038/nature07205 18650914

[59] Pikarsky E Porat RM Stein I Abramovitch R Amit S Kasem S et al. NF-κB functions as a tumour promoter in inflammation-associated cancer. Nature (2004) 431:461–6. 10.1038/nature02924 15329734

[60] Bravo SB Pampin S Cameselle-Teijeiro J Cerneiro C Dominguez F Barreiro F. TGF-β-induced apoptosis in human thyrocytes is mediated by p27kip1 reduction and is overridden in neoplastic thyrocytes by NF-κB activation. Oncogene (2003) 22:7819–30. 10.1038/sj.onc.1207029 14586408

[61] De Falco V Carlomagno F Li HY Santoro M The molecular basis for RET tyrosine-kinase inhibitors in thyroid cancer. Best Pract Res Clin Endocrinol Metab. (2017) 31:307–18. 10.1016/j.beem.2017.04.013 28911727 PMC5624797

[62] Acquaviva G Visani M Repaci A Rhoden KJ de Biase D Pession A. Molecular pathology of thyroid tumours of follicular cells: a review of genetic alterations and their clinicopathological relevance. Hystopathology (2018) 72:6–31. 10.1111/his.13380 29239040

[63] Ludwig L Kessler H Wagner M Hoang-Vu C Dralle H Adler G. Nuclear factor-κB is constitutively active in C-cell carcinoma and required for RET-inducced transformation. Cancer Res. (2001) 61:4526–35. 11389085

[64] Spitschak A Meier C Kowtharapu B Engelmann D Pützer BM. MiR-182 promotes cancer invasion by linking RET oncogene activated NF-κB to loss of the HES1/Notch1 regulatory circuit. Mol Cancer (2017) 16:24. 10.1186/s12943-016-0563-x. 28122586 PMC5267421

[65] Borrello MG Alberti L Fischer A Degl'Innocenti D Ferrario C Gariboldi M. Induction of a proinflammatory program in normal human thyrocytes by the RET/PTC1 oncogene. Proc Natl Acad Sci USA (2005) 102:14825–830. 10.1073/pnas.0503039102 16203990 PMC1253545

[66] Visconti R Cerutti J Battista S Fedele M Trapasso F Zeki K Expression of the neoplastic phenotype by human thyroid carcinoma cell lines requires NF-κB p65 protein expression. Oncogene (1997) 15:1987–94. 10.1038/sj.onc.1201373 9365245

[67] Palona I Namba H Mitsutake N Starenki D Podcheko A Sedliarou I. BRAFV600E promotes invasiveness of thyroid cancer cells through nuclear factor-κB activation. Endocrinology (2006) 147:5699–707. 10.1210/en.2006-0400 16959844

[68] Kato Y Ying H Zhao L Furuya F Araki O Willingham MC. PPARγ insufficiency promotes follicular thyroid carcinogenesis via activation of the nuclear factor-κB signaling pathway. Oncogene (2006) 25:2736–47. 10.1038/sj.onc.1209299 16314832

[69] Guigon CJ Zhao L Willingham MC Cheng S-Y. PTEN deficiency accelerates tumour progression in a mouse model of thyroid cancer. Oncogene (2009) 28:509–17. 10.1038/onc.2008.407 18997818 PMC3457778

[70] Liu J Brown RE Morphoproteomic confirmation of an activated nuclear factor-?B p65 pathway in follicular thyroid carcinoma. Int J Clin Exp Pathol. (2012) 5:216–23. 22558476 PMC3341672

[71] Pyo JS Kang G Kim DH Chae SW Park C Kim K. Activation of nuclear factor-κB contributes to growth and aggressiveness of papillary thyroid carcinoma. Pathol Res Pract. (2013) 209:228–32. 10.1016/j.prp.2013.02.004 23528368

[72] Li W Ming H Sun D Li W Wang D Zhang G. The relationship between BRAFV600E NF-κB and TgAb expression in papillary thyroid carcinoma. Pathol Res Pract. (2017) 213:183–8. 10.1016/j.prp.2016.12.022 28214213

[73] Li X Abdel-Mageed AB Mondal D Kandil E The nuclear factor-κB signaling pathway as a therapeutic target against thyroid cancers. Thyroid (2013) 23:209–18. 10.1089/thy.2012.0237 23273524

[74] Starenki D Namba H Saenko V Ohtsuru A Yamashita S. Inhibition of nuclear factor-κB cascade potentiates the effect of a combination treatment of anaplastic thyroid cancer cells. J Clin Endocrinol Metab. (2004) 89:410–8. 10.1210/jc.2003-031216 14715879

[75] Zhu W Ou Y Li Y Xiao R Shu M Zhou Y A small-molecule triptolide suppresses angiogenesis and invasion of human anaplastic thyroid carcinoma cells via down-regulation of the nuclear factor-κB pathway. Mol Pharmacol. (2009) 75:812–9. 10.1124/mol.108.052605 19158360

[76] Cras A Politis B Balitrand N Darsin-Bettinger D Boelle PY Cassinat B. Bexarotene via CBP/p300 induces suppression of NF-κB–dependent cell growth and invasion in thyroid cancer. Clin Cancer Res. (2012) 18:442–53. 10.1158/1078-0432.CCR-11-0510 22142826

[77] Vasudevan KM Gurumurthy S Rangnekar VM Suppression of PTEN expression by NF-κB prevents apoptosis. Mol Cell Biol. (2004) 24:1007–21. 10.1128/MCB.24.3.1007-1021.2004 14729949 PMC321419

[78] Reale C Zotti T Scudiero I Vito P Stilo R. The NF-κB family of transcription factors and its role in thyroid physiology. Vitam Horm. (2018) 106:195–210. 10.1016/bs.vh.2017.05.003 29407436

